# Integrating molecular and physiological approaches to quantify genetic controls for wheat development and improve phenotyping

**DOI:** 10.1093/jxb/erag278

**Published:** 2026-06-05

**Authors:** Hamish Brown, John McCallum, Paul Johnston, Meeghan Pither-Joyce, Richard Macknight, Neil Huth, Derrick Moot, Bangyou Zheng, Zhigan Zhao, James Hunt, Enli Wang

**Affiliations:** The New Zealand Institute for Bioeconomy Science, Christchurch 7608, New Zealand; The New Zealand Institute for Bioeconomy Science, Christchurch 7608, New Zealand; Department of Biochemistry, University of Otago, Dunedin 9054, New Zealand; The New Zealand Institute for Bioeconomy Science, Christchurch 7608, New Zealand; The New Zealand Institute for Bioeconomy Science, Christchurch 7608, New Zealand; Department of Biochemistry, University of Otago, Dunedin 9054, New Zealand; CSIRO Agriculture and Food, Forest Hill, Queensland 4342, Australia; Faculty of Agriculture and Life Sciences, Lincoln University, Canterbury 7647, New Zealand; CSIRO Agriculture and Food, St Lucia, Queensland 4067, Australia; CSIRO Agriculture and Food, Black Mountain, Australian Capital Territory 2601, Australia; School of Agriculture, Food and Ecosystem Sciences, University of Melbourne, Parkville, Victoria 3010, Australia; CSIRO Agriculture and Food, Black Mountain, Australian Capital Territory 2601, Australia; University of Sydney, Australia

**Keywords:** Final leaf number, flowering time, gene expression, phenology, photoperiod, *Triticum aestivum*, vernalization, *VRN* genes

## Abstract

Disentangling genotype×environment (G×E) controls of flowering time requires phenotypes that link molecular regulation, developmental physiology, and environment. Here, we integrated time-resolved measurements of apical development, final leaf number, and expression of the flowering-time genes *VRN1*, *VRN2*, and *VRN3* across contrasting temperature and photoperiod regimes in six wheat genotypes spanning a wide range of developmental sensitivities. By combining controlled-environment phenotyping with concurrent gene-expression profiling, we show that environmentally driven variation in final leaf number is coherently explained by shifts in the timing of key apical transitions and associated *VRN* gene-expression dynamics. These integrated datasets were used to parameterize and interrogate the Cereal Anthesis Molecular Phenology (CAMP) model, enabling direct comparison between observed foliar gene-expression time courses and modelled gene activity. While overall developmental responses were well captured by the model, systematic differences between observed and modelled gene-expression patterns highlight the importance of distinguishing foliar expression from apical regulatory activity, as well as differences in temporal scaling. Building on this framework, we present a phenotyping protocol based on final leaf number responses to defined temperature and photoperiod treatments that delivers unconfounded developmental phenotypes explicitly linked to underlying genetic regulation.

## Teaser

Integrated phenological and gene-expression time courses across environments enabled robust phenotyping and validation of a molecularly grounded flowering model for wheat.

## Introduction

The key phenotype for matching cereal genotypes best suited to a given environment is anthesis date. A substantial amount of effort is invested in understanding the genetic controls of this phenotype. A measure of when anthesis occurs in days after sowing does not provide a stable phenotype because different genotypes respond to temperature and photoperiod (Pp) in different ways, so days after sowing interacts with location and sowing date. Process models are useful tools for quantifying genotype by environment by management (G×E×M) responses and providing genotype-specific coefficients that can be used as stable phenotypes giving more power in genomic analyses (Wang *et al.*, 2019). The ‘Kirby framework’ (Brown *et al.*, 2013) provides a useful structure for capturing the G×E×M of anthesis timing by representing the underlying physiological mechanisms. Specifically, flag leaf appearance always precedes anthesis, and prediction of its timing is critical. The time of flag leaf appearance depends on the number of main-stem leaves produced, referred to as final leaf number (FLN), and the thermal time duration that is required for each leaf to appear (called phyllochron). Phyllochron, quantified as °C days per leaf, changes with crop development, photoperiod, and genotype (Slafer and Rawson, 1997; Baumont *et al.*, 2019; Bloomfield *et al.*, 2023).

The FLN of a wheat crop depends on the prevailing environmental conditions and the genotype’s sensitivity to photoperiod and cold temperatures (Brooking *et al.*, 1995; Robertson *et al.*, 1996; Brooking and Jamieson, 2002; Gonzalez *et al.*, 2002; Bloomfield *et al.*, 2023). A complete quantitative understanding of the response of FLN to genotype and environment is still lacking. Brown *et al.* (2013) attempted to progress this understanding, proposing an integrated model, combining molecular pathway knowledge of development processes with a deterministic phenology model. Herein the conceptual model described by Brown *et al.* (2013) is referred to as the Cereal Anthesis Molecular Phenology (CAMP) model. The CAMP model provides a quantitative framework in which its parameters can represent expression rates of virtual genes that may ultimately be linked to marker, single nucleotide polymorphism, quantitative trait loci, or genome data to enable estimation of the anthesis date of any known genotype in any defined environment. The temporal patterns of gene expression predicted in CAMP are not of absolute concentrations, rather expression relative to an arbitrary level required to trigger an event. The algorithms for predicting their expression were developed using expected patterns of gene expression required to reach key phenological events. However, they have not been validated with experimental material where temporal patterns of gene expression and observations of phenological events have been observed simultaneously.

This paper firstly updates the conceptualization of the CAMP model based on latest understanding. Then, the mechanistic framework encapsulated in CAMP was used to design a set of developmental phenotypes and specify a set of environmental treatments needed to quantify these. Next is a description of results from an experiment where six diverse genotypes of wheat were grown with different temperature and photoperiod treatments and their *VRN* gene expression and phenology throughout the vegetative and early reproductive phases were measured. Finally, these data are compared with the predictions of CAMP to verify its concepts of gene expression and genetic control.

### CAMP model general description

A full description of the CAMP model algorithms, including the numerical scheme used to derive model parameters from developmental phenotypes based on final leaf number (FLN), is provided in the Supplementary Appendixes S1, S2. This section presents an updated conceptual overview of the model first proposed by Brown *et al.* (2013) and subsequently refined based on the work reported in this study and an allied paper (Wang *et al.*, 2026). This overview provides a description of how CAMP links environmental cues, gene activity, and developmental progression prior to introducing technical detail.

**Fig. 1.**
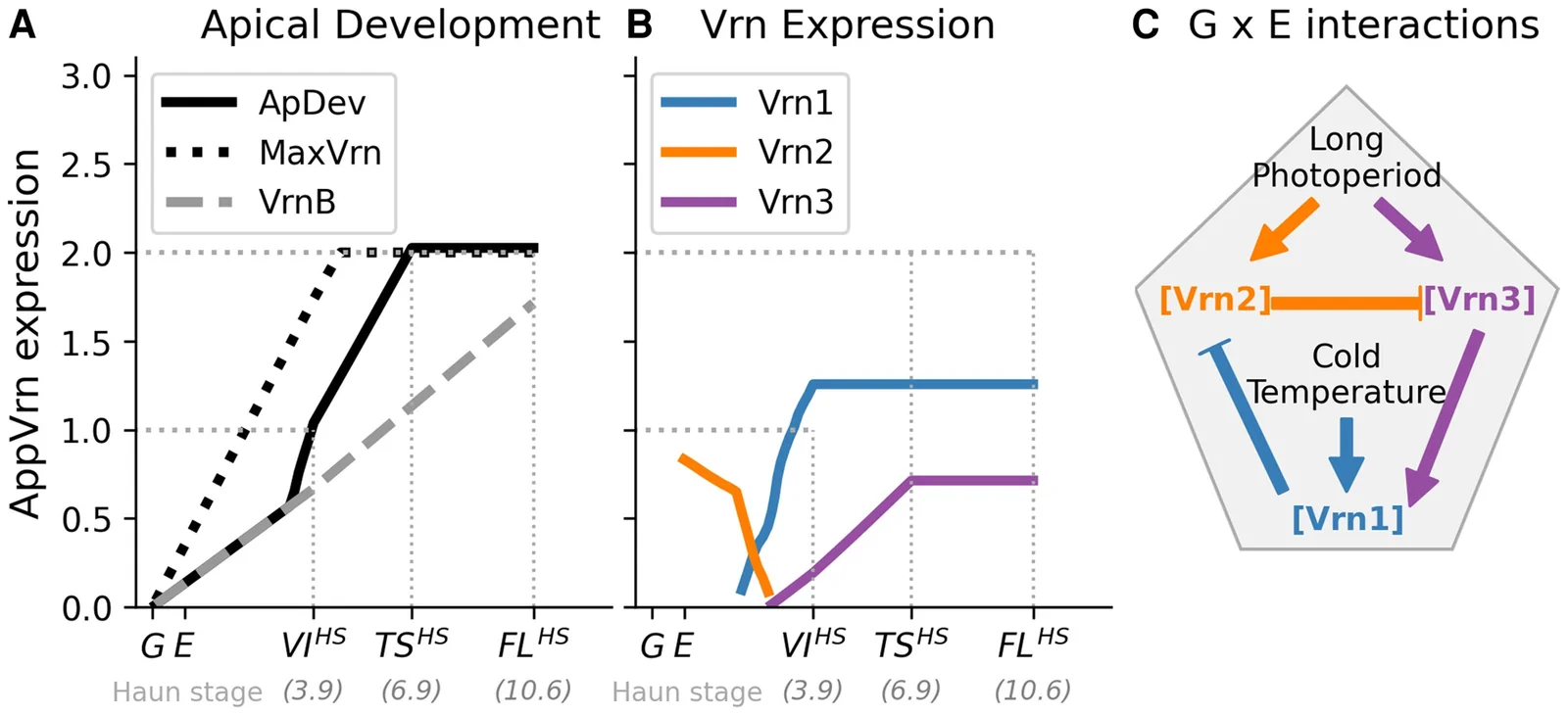
An example of the CAMP models time series predictions of apical development (A), Vrn gene expression (B) and gene and environment interactions (C). (A) Representation of apical development (ApDev) in CAMP as a bounded accumulation process with respect to Haun stage (HS). Developmental progress is constrained by a maximum rate (MaxVrn) and a base rate (VrnB), illustrating that CAMP represents relative developmental effect rather than direct gene expression on morphology. (B) Idealized relative activity of the major flowering-time genes *Vrn1*, *Vrn2*, and *Vrn3* across developmental progression, shown on the same HS scale. Vertical dashed lines indicate the HS timing of vernal induction (VI^HS^), terminal spikelet (TS^HS^), and flag leaf appearance (FL^HS^). Stage labels on the *x*-axis denote developmental events; bracketed values beneath axis ticks indicate representative HS timings for the example simulation and are provided for orientation only. (C) Schematic overview of genotype×environment (G×E) interactions assumed in CAMP, showing the qualitative regulatory effects of temperature and photoperiod on Vrn gene activity. Cold temperature promotes Vrn1, while long photoperiod promotes Vrn2 and Vrn3, with Vrn2 antagonizing Vrn3. Panels together illustrate how environmental cues influence gene activity within developmental bounds to regulate the timing of key phenological stages.

As a note on nomenclature, *VRN* represents the flowering time genes and up-regulation in the plant; where Vrn is used, it represents a model variable name. Figure 1 provides a representation of the CAMP framework. The model’s conceptual basis uses an apical development index (ApDev) to predict progression through early phenological stages (Fig. 1A). The level of ApDev is constrained between a lower base vernalization (VrnB) and upper maximum vernalization (MaxVrn) and is modulated by changes in the activity of key flowering-time genes as the crop develops (Fig. 1B). Environmental signals associated with temperature and photoperiod influence this gene activity, giving rise to genotype×environment (G×E) effects on the timing of key developmental transitions (Fig. 1C).

The timing of flag leaf appearance is central to the model, and CAMP splits this period into four phases. These are:

- an emerging phase, from seed imbibition to emergence, during which exposure to low temperature may reduce FLN;
- a vegetative phase, from emergence to vernal induction (VI), during which exposure to low temperature may reduce FLN and photoperiod may either increase or decrease FLN depending on genotype;
- an early reproductive phase, from VI to terminal spikelet (TS), during which long photoperiod typically accelerates development and may reduce FLN;
- a stem extension phase, from TS to flag leaf expansion, during which FLN is fixed and remaining leaves emerge.

The wheat crop exhibits vernal (spring-like) behaviour in the early reproductive phase where development accelerates in response to lengthening photoperiod. During the early vegetative phase, photoperiod responses are also evident, and experimental observations indicate that these responses may act in an opposing direction compared with later developmental phases (Brooking and Jamieson, 2002; Allard *et al.*, 2012). Explicitly separating these phases is therefore necessary to effectively disentangle environmental responses and their genetic control.

With the importance of the vernal induction and terminal spikelet transitions established, CAMP represents their timing through the combined effects of *VRN* gene activity. Three genes and their interaction (Fig. 1C), play central roles in this process: *VRN1* is a meristem identity gene that promotes spikelet differentiation when expressed above a threshold level (Trevaskis, 2010); *VRN3* encodes the florigen signal and promotes *VRN1* expression at the apex (Yan *et al.*, 2006); *VRN2* blocks the action of *VRN3* and is down-regulated by *VRN1* (Li and Dubcovsky, 2008). *VRN2* represses floral development by antagonizing *VRN3*, thereby increasing the *VRN1* activity required to reach vernal induction (Yan *et al.*, 2006). Once *VRN2* is down-regulated, *VRN3* may be further up-regulated during the early reproductive phase, accelerating progress toward terminal spikelet.

To reach vernal induction in CAMP, Vrn1 expression must first increase sufficiently to suppress Vrn2, allowing Vrn3 activity (Fig. 1B). The combined promotive effects of Vrn1 and Vrn3 must then exceed a developmental threshold to trigger vernal induction (Fig. 1A). Base expression levels and sensitivities of these genes differ among genotypes and are modulated by environment: *VRN1* may be up-regulated by exposure to low temperature, while *VRN2* and *VRN3* are regulated by photoperiod, such that short photoperiod accelerates progress toward vernal induction by reducing Vrn2, whereas long photoperiod promotes Vrn3 once Vrn2 repression is relieved.

*VRN1* is up-regulated regardless of temperature in spring genotypes, but up-regulated faster at lower temperatures in winter genotypes (Trevaskis *et al.*, 2007; Diaz *et al.*, 2012). As such, linking genotypic data to Vrn1 expression coefficients gives a mechanism for quantifying low-temperature responses in the timing of vernal induction. *VRN2* is up-regulated in response to long photoperiod in some genotypes, but absent in the genome of others (Yan *et al.*, 2004; Distelfeld and Dubcovsky, 2010; Li *et al.*, 2011). Thus, linking genotypic data to Vrn2 expression coefficients gives a mechanism for quantifying short photoperiod responses in the timing of vernal induction. *VRN3* is up-regulated in response to long photoperiods and the extent of response is dependent on photoperiod genes (Yan *et al.*, 2006; Sasani *et al.*, 2009; Kitagawa *et al.*, 2012; Shaw *et al.*, 2012). Therefore, linking genotype data to Vrn3 expression coefficients gives a mechanism for quantifying long photoperiod responses in the timing of vernal induction and terminal spikelet. Linking gene expression to the timing of phenological events and further linking phenology to FLN provides a framework to understand G×E at the molecular and physiological levels. It also allows us to design methods to quantify G×E for the different developmental phenotypes of wheat.

It is important to note that CAMP ascribes different roles to VrnB and Vrn1, base development and cold response respectively, but both fulfil the same fundamental requirement of up-regulation to initiate a change in floral identity of the apex. As such, we assume VrnB as modelled in CAMP equated to *VRN1* expression as observed in wheat leaf tissue.

The FLN is set when the terminal spikelet formed and the crop’s phyllochron is used to predict leaf appearance (quantified by Haun stage, HS) to determine when flag leaf emergence occurs. Temperature is a key driver of timing of flag leaf emergence, independent of photoperiod and cold temperature effects on FLN, because it drives the development of HS. This effect is captured in CAMP by deriving apparent gene expression and the timing of vernal induction and terminal spikelet relative to Haun stage (VI^HS^ and TS^HS^) rather than days. The duration from flag leaf emergence to anthesis is calculated from a genotype specific thermal time target and photoperiod sensitivity.

### Quantification of developmental phenotypes

#### Environmental treatments

The analysis scheme developed below uses measurements of FLN of wheat genotypes exposed to cold (C) or warm (W) temperature treatments at the start of their development cycle. These are applied in factorial combination with long (L) and short (S) photoperiod from emergence. This requires defining environmental conditions that achieve complete or negligible responses to low temperature and that ensure long and short photoperiods lie close to the upper and lower response thresholds. To ensure photoperiod responses are not confounded by low-temperature responses and vice versa, a cold treatment should be applied as early as possible. Wheat begins to accumulate low-temperature response as soon as the seed is imbibed. This can be saturated by holding imbibed seed at 1 °C in the dark for >90 d (Brooking and Jamieson, 2002; Dixon *et al.*, 2019).

Although vernalization responses are often described on a calendar-time basis, for which temperatures near 6 °C maximize the daily rate of response, development in this study is quantified relative to Haun stage. When expressed on an HS basis, lower temperatures (1 °C) are optimal because they substantially reduce leaf appearance rate while allowing vernalization to accumulate. At temperatures near 6 °C, plants would have reached approximately HS 3 by the time vernalization was complete, confounding early developmental progression with exposure to cold. Using 1 °C therefore ensured completion of vernalization while maintaining plants at very early developmental stages, maximizing resolution of vernalization effects relative to HS.

The process of germination and emergence takes approximately 100 °C days, so under these conditions the cold temperature response could be saturated before emergence, which is the approach used for the C treatments in this study. Alternatively, sowing seed into pots maintained at 6 °C can also achieve complete vernalization at or soon after HS 2 (Bloomfield *et al.*, 2023). Temperatures of up to 15 °C have been shown to induce reductions in FLN (Brooking and Jamieson, 2002), so plants in the W treatment were maintained at day and night temperatures >20 °C. Plants in the C treatment were moved to W conditions at emergence. For wheat, photoperiod responses are well approximated by a linear increase from a base of 8 h to an upper threshold of 16 h (Jamieson *et al.*, 1998), and therefore these values were used to represent the S and L treatments, respectively.

#### Minimum leaf number

**Fig. 2.**
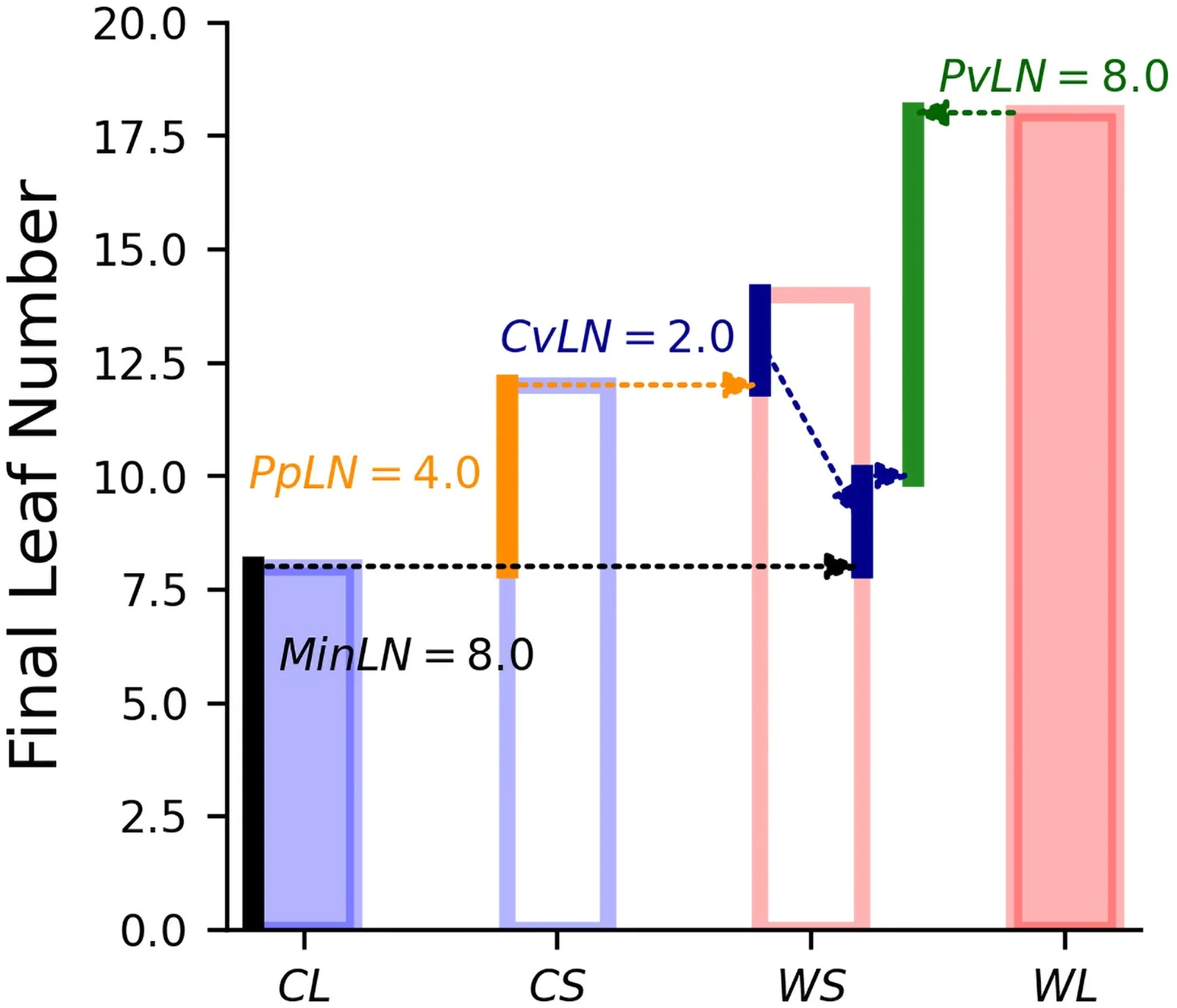
Schematic illustration of the derivation of developmental phenotypes from final leaf number (FLN) measurements using selected controlled-environment treatments for the winter wheat cultivar ‘Batten’, adapted from Brooking and Jamieson (2002). Blue and red bars represent FLN measured with (C) and without (W) cold treatment during the emerging phase, respectively, followed by growth under long (L) or short (S) photoperiod. Minimum leaf number (MinLN) is defined as FLN under CL conditions. Photoperiod sensitivity (PpLN) is calculated as the increase in FLN between CS and CL. Cold vernalization sensitivity (CvLN) is calculated as the additional increase in FLN under WS after accounting for MinLN and PpLN. Photoperiod vernalization sensitivity (PvLN) is calculated as the difference in FLN between WL and WS after accounting for MinLN and CvLN. Values shown illustrate the difference scheme; PvLN may be positive or negative depending on genotype.

If a genotype receives cold treatment, vernal induction will be reached at its earliest possible time and, if grown in long-day conditions (CL), it will also progress to terminal spikelet at the earliest possible time. Thus, for plants grown in CL conditions, the observed FLN will represent its minimum (minimum leaf number, MinLN) and can be used as a measure of earliness *per se* for the genotype (Fig. 2):


(1) $$\mathrm{MinLN}=CL^{\mathrm{FLN}}$$

#### Photoperiod sensitivity

If a genotype receives cold treatment and is grown in short-day conditions (CS) its FLN may increase (Fig. 2). Downregulation of *VRN3* will extend the duration of the early reproductive phase. Expression of *VRN3* decreases with shortening photoperiod, and the extent of this decrease depends on genotype (Yan *et al.*, 2006; Sasani *et al.*, 2009; Kitagawa *et al.*, 2012; Shaw *et al.*, 2012). When *VRN3* expression is minimized under short photoperiod, the early reproductive duration will reach a maximum. Therefore, the photoperiod sensitivity of a genotype (PpLN) may be quantified as the difference in FLN between the long and short photoperiod treatments for cold treated genotypes (Fig. 2):


(2) $$\mathrm{PpLN}=CS^{\mathrm{FLN}}-CL^{\mathrm{FLN}}$$

#### Cold vernalization sensitivity

When a crop is grown in continuously warm conditions (W), the duration of its vegetative phase and FLN may increase. However, the extent of the FLN response interacts with photoperiod. Considering the warm short-day treatment (WS), FLN will be increased by response to short photoperiod in the early reproductive phase but may be further extended by lack of cold exposure prior to vernal induction. Some alleles of VRN1 will only up-regulate *VRN1* expression in the cold (Trevaskis *et al.*, 2007; Diaz *et al.*, 2012). Such genotypes will be slower to reach vernal induction if warm conditions are imposed and any increase in FLN beyond that already attributed to PpLN provides a quantification of the cold vernalization sensitivity (CvLN) of the genotype (Fig. 2):


(3) $$\mathrm{CvLN}=WS^{\mathrm{FLN}}-\mathrm{MinLN}-\mathrm{PpLN}$$

#### Photoperiod vernalization sensitivity

When a genotype is grown in warm conditions with long photoperiod (WL), the duration of its vegetative phase and subsequent FLN may be increased or decreased relative to the WS treatment depending on the relative activity of the photoperiod sensitive genes (*VRN2* and *VRN3*). The photoperiod vernalization sensitivity (PvLN) of the vegetative phase is quantified as (Fig. 2):


(4) $$\mathrm{PvLN}=WL^{\mathrm{FLN}}-\mathrm{MinLN}-\mathrm{CvLN}$$

This may be a positive or negative value. In the case of a genotype that actively expresses *VRN2* under long-day conditions, this will block *VRN3* and delay vernal induction causing FLN to increase and give a positive PvLN. This response has been described as short-day vernalization (Brooking and Jamieson, 2002; Allard *et al.*, 2012). In the case of a genotype with low *VRN2* activity, *VRN3* may be up-regulated during the vegetative phase, which will make vernal induction earlier under long-day conditions, reducing FLN and giving a negative number for PvLN.

## Materials and methods

### Plant material and culture

The six genotypes selected for this study span a diverse range of vernalization and photoperiod sensitivities observed in the field. The Batten near isogenic lines (winter and spring) have different vernalization sensitivities (Brooking and Jamieson, 2002) while Otane is a true spring wheat with no vernalization response. ‘Saracen’ is a cultivar with intermediate vernalization requirement; ‘Amarok’ is a winter wheat cultivar with low photoperiod sensitivity, and ‘CRWT153’ is a breeding line that consistently displays very late anthesis in field trials. The developmental phenotypes of these genotypes observed in the field were consistent with the allelic composition for *VRN* and *PPD* genes revealed by published markers (Table 1).

**Table 1.** Allelic composition of wheat genotypes used in controlled environment phenology studies described in this paper

		Genotype					
Marker (locus: assay)	Reference	Otane	Batten spring	‘Saracen’	Batten winter	‘Amarok’	‘CRWT153’
*VRN1*: F/R marker	Yan *et al.* (2004)	A1a (spring deletion)	A1a (spring deletion)	A1 (wild type—winter)	A1 (wild type—winter)	A1 (wild type—winter)	A1 (wild type—winter)
*VRN-B1*: deletion marker (TDB assay)	Fu *et al.* (2005)	Spring deletion	Non-deletion	Non-deletion	Non-deletion	Non-deletion	Non-deletion
*PPD-D1*: F/R1–R2 marker	Beales *et al.* (2007)	Insensitive	Sensitive	Sensitive	Sensitive	Insensitive	Sensitive
*FT-A1*: allele-sequencing marker (PJF3)	Bonnin *et al.* (2008)	h4	h1	h3	h1	h3	Not measured
*FT-D1*: allele-sequencing marker (PJF2)	Bonnin *et al.* (2008)	h2	h1	h2	h1	h2	Not measured
*VRN2* (*VRN-A1/B1/D1*): F1–R2 marker	Zhu *et al.* (2014)	ABD (no null alleles)	ABD (no null alleles)	ABD (no null alleles)	ABD (no null alleles)	ABD (no null alleles)	ABD (no null alleles)

Gene symbols (e.g. *VRN1*, *VRN-B1*, *PPD-D1*, *FT-A1*, *FT-D1*, *VRN2*) are shown in italics following standard wheat gene nomenclature. Non-italic text denotes the type of molecular assay used to genotype each locus, including PCR-based markers (e.g. F/R primer pairs), deletion markers, or allele-sequencing assays. Marker names therefore comprise the gene locus followed by the assay type, separated by a colon. Allelic states are described as ‘spring’, ‘winter’, ‘deletion’, ‘non-deletion’, or haplotype classes (e.g. h1, h2, h3, h4), as defined in the cited references. Where indicated, ‘insensitive’ and ‘sensitive’ refer to photoperiod response at the *PPD-D1* locus. ‘Not measured’ indicates genotypes for which marker data were unavailable.

Plants were grown in a Conviron growth room (Controlled Environments Ltd, Manitoba, Canada) at the Lincoln University Biotron facility (Shi *et al.*, 2011). Growth rooms were run with a constant 16 h photoperiod and 25/20 °C light/dark temperature (L) or an 8 h photoperiod and 25/22 °C light/dark temperature (S). Lights were switched on in three separate banks at 30 min intervals at the beginning of the photoperiod and produced photosynthetic photon flux density of 1150 μ mol m^2^ s^−1^ when all three banks were on. Lights were switched off one bank at a time in 30 min intervals at the end of the photoperiod. The growth room had mirrored walls and a well-mixed atmosphere to provide even environmental conditions. Pots of three to six plants of the same genotype were established in bark and soil-based potting mix. Pots were assembled on 1.2 m high benches with raised sides and a central drainage hole. Up to 600 pots could be included with this layout, which provided ample space for three replicates and up to 12 repeat samplings of each treatment. Pots were laid out in a random block design with three replicates per environmental treatment to account for any possible variation in air flow or radiation.

For warm-temperature treatments (W), six seeds were sown in each pot and covered with a 15 mm layer of vermiculite. Vermiculite was used instead of potting mix because it offers little resistance to coleoptile extension, ensuring even emergence. It also has a high albedo that limits radiant heating of the pots to assist with keeping the apex temperature of the wheat consistent with air temperature.

For the cold-temperature treatments (C), seeds were sterilized by immersion in 4% sodium hypochlorite and stirred for 30 min. Seeds were then rinsed with reverse osmosis water and transferred to sterile Petri dishes containing moist filter paper. Petri dishes were put inside a polystyrene box to exclude light and maintain a stable temperature. This box was kept in a refrigerator (1±0.5 °C) for 90 d and dishes were checked weekly. Re-wetting of filter paper with reverse osmosis water was undertaken as needed and any seeds showing signs of fungal growth were removed. A HOBO temperature logger (Onset Computer Corp., Bourne, MA, USA) was kept in the box to record hourly temperature. At the completion of this treatment, seeds had germinated with a 1–5 mm coleoptile and 10–20 mm roots visible. These plants were carefully teased apart, transplanted into 1 litre pots (three to six plants per pot) and covered with a 15 mm layer of vermiculite (as for the W treatments). From this time on, plants were grown in the same conditions as the W treatment.

Pots were watered with a watering can until 1 week after emergence. At this time, an irrigation dripper was installed in each pot. Moisture sensors were also installed in four sentinel pots (pots with well-established plants). An irrigation control system using a CR1000 data logger (Campbell Scientific, Logan, UT, USA) turned on all drippers for 1 min each time the mean water content of the sentinel pots reached a specified trigger point. Any water draining from the tables was plumbed into the growth chamber’s condensation collection system. This method enabled the continual irrigation of many pots without wetting the foliage of the plants, so avoiding foliar disease problems. Beyond 8 weeks, plants showed evidence of nutrient stress, which suggests their growth demands had exceeded the supply of nutrients in the potting mix. From this point, each pot received 100 ml of Hoagland’s solution (Hoagland and Arnon, 1950) weekly.

### Plant sampling

Samples were taken at weekly intervals. The timing of the photoperiod was set so the lights went out at 10.00 am. This allowed time to take leaf samples for RNA analysis at the end of the light period when gene expression was determined to be most representative of the treatments (see Supplementary Appendix S3). For RNA isolation, the most recent fully expanded leaf was cut (using sharp scissors) at its mid length, and immediately ∼5×1 mm strips (∼1 cm^2^, ∼25 mg) were cut from the basal end of the sampled leaf directly into a 1 ml tube containing 300 µl of DNA/RNA Shield (Zymo Research Ltd). Care was taken to completely submerge the leaf tissue in the DNA/RNA shield. Samples were stored at −20 °C for later RNA isolation. One pot was taken from each replicate (three pots per treatment) giving 9–18 plants per treatment for dissection at each sampling date. Three leaves were taken for RNA analysis as described above. The remainder of the plants were removed from their pots, the number of fully expanded main-stem leaves (those with ligules visible) were counted, and the length of the most recently fully expanded leaf and the current expanding leaf was measured for retrospective calculation of HS (Brooking and Jamieson, 2002). The irrigation method meant the canopies remained dry and senesced leaves did not decompose, allowing the number of emerged leaves to be determined accurately. Each plant was then dissected under a binocular microscope to observe and count the number of un-emerged leaves and the number of primordia on the apex that were yet to differentiate and emerge (Brooking and Jamieson, 2002). Measurements were continued until seed heads emerged. At this time, any remaining pots (6–12 pots giving 30–60 plants per treatment) were destructively sampled to measure FLN and the number of spikelets on the spike.

### Observed timings of vernal induction and terminal spikelet stages

**Fig. 3.**
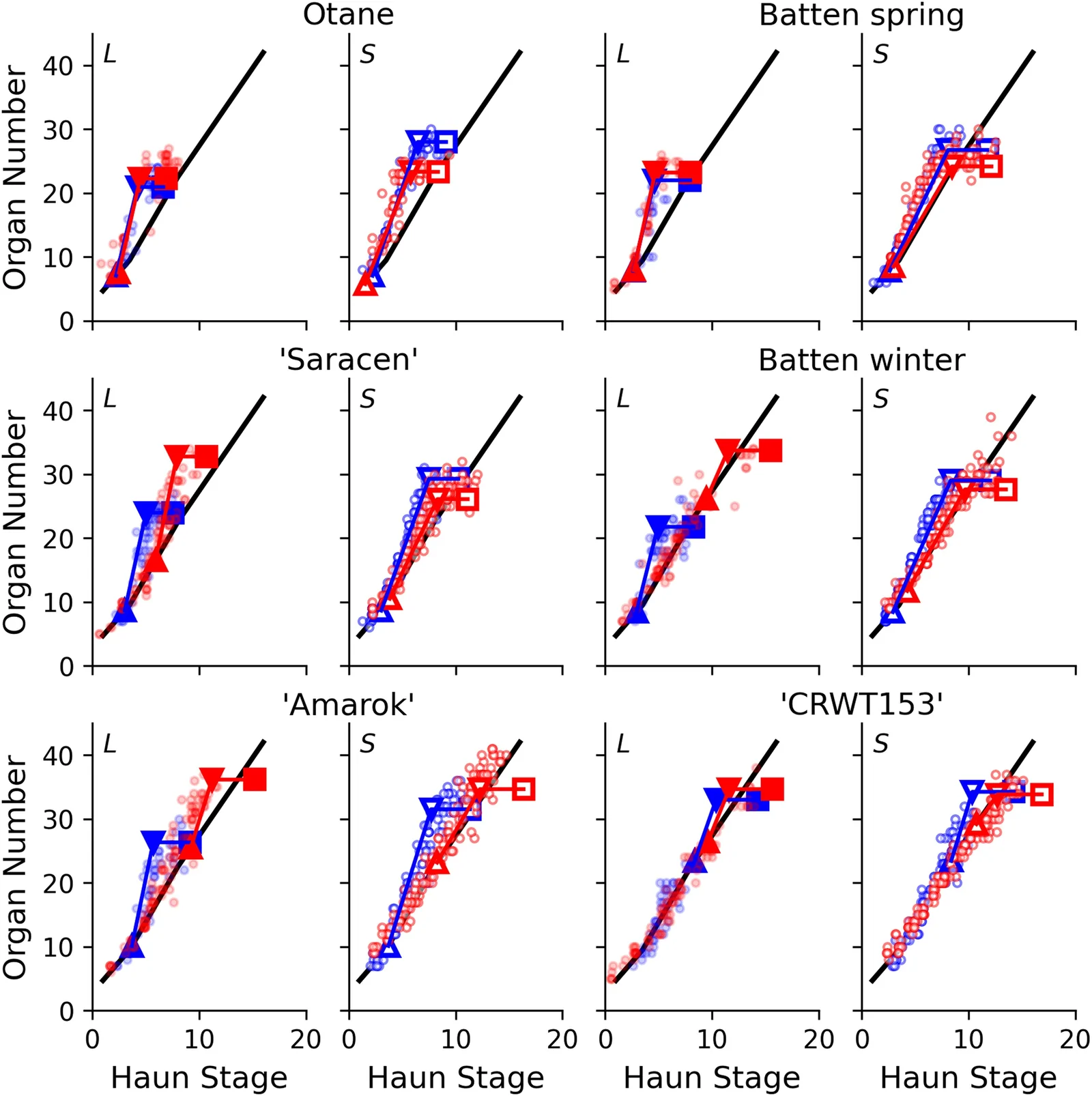
The relationship between total main-stem organ number (leaves, undifferentiated primordia, and spikelets) and Haun stage (HS; measure of main-stem leaf appearance) for different genotypes of wheat grown with (blue) or without (red) cold treatment in combination with long (filled markers) or short (open markers) photoperiod. The large squares are the organ number observed at final sampling (*y*-axis), plotted against observed final leaf number (FLN; *x*-axis). The large downward pointing triangles have the same *y*-value plotted against the HS timing of terminal spikelet (TS^HS^) predicted by the CAMP parameter scheme using the observed FLN. The large upward pointing triangles are the HS timing of vernal induction (VI^HS^) calculated from the CAMP parameter scheme (*x*-value) plotted against the organ number produced at the base vegetative rate (displayed by the black line) at VI^HS^. The small circles represent observations of total organ number at different HS from repeat destructive samplings. The black line represents the relationship that vegetative plants are expected to conform to (Brown *et al.*, 2013). Treatments are denoted as CL (cold + long photoperiod), CS (cold + short photoperiod), WS (warm + short photoperiod), and WL (warm + long photoperiod).

The timings of VI and TS were quantified in terms of the HS at which they occurred and are superscripted with HS to show this. During the vegetative phase, wheat produces primordia at a rate about double that of ligule appearance (Brooking and Jamieson, 2002). This rate increases when the crop reaches VI and remains elevated until TS. As such, the HS timings of these events can be identified as break points on a plot of total organ number (leaves, undifferentiated primordia, and spikelets) against HS at each destructive sampling (Fig. 3).

### RNA isolation and quantification

Frozen samples were allowed to equilibrate to room temperature. A leaf homogenate was made by adding 0.5 mm-diameter glass beads to the sample tube and grinding with the flat end of a 0.5 ml microtube pestle. After centrifuging at 2000*×g* for 10 min, 60 µl of supernatant was transferred to a new 0.5 ml deep well plate and an equal volume of RNA Lysis Buffer (Zymo Research) was added. Total RNA was isolated using the ZR-96 Quick RNA Kit (Zymo Research) according to manufacturer’s instructions and serially eluted with 1×25 µl and 1×10 µl DNase/RNase free water preheated to 95 °C. RNA concentration was determined using a Victor^3^ Multilabel Plate Reader (Perkin Elmer) and the Quant-IT RiboGreen RNA quantification kit (Thermo Fisher Scientific).

### Two-step quantitative real-time PCR

Leaf tissue samples were pooled for three plants from each sample pot (three replicate pots per treatment), and two technical replicates were conducted on each of the three biological replicates. Reverse transcription was carried out with 100 ng of total RNA in a volume of 20 μl using Maxima First Strand cDNA Synthesis Kit for quantitative real-time PCR (RT-qPCR), with dsDNase (Thermo Fisher Scientific) according to the manufacturer’s instructions. A replicate template plate for each primer set was made by diluting first-strand cDNA 5-fold with double distilled H_2_O and transferring 3×5 µl aliquots (technical replicates) into PCR plates. An identical sample was loaded into every template plate as an inter-run calibrator and plates were stored at −80 °C. RT-qPCR was performed in 10 µl reaction volumes in 384-well plates on the LightCycler 480 System (Roche Life Science) using SYBR Green detection and Luminaris Color HiGreen High ROX qPCR Master Mix (Thermo Fisher Scientific) according to the manufacturer’s instructions. The cycling conditions were: 2 min hold at 50 °C, 10 min hold at 95 °C, followed by 40 amplification cycles of 15 s at 95 °C, 1 min hold at 60 °C. After completion of qPCR, melt curve analysis was carried out on the amplicons using the default settings of the real-time PCR instrument.

### Primer set validation

Published primer sets targeting *VRN1* (WAP1), *VRN2* (ZCCT1), *VRN3* (WFT), and the housekeeping genes *ubiquitin* (Shimada *et al.*, 2009) and *Ta54227* (Paolacci *et al.*, 2009) were initially evaluated. Primer performance was assessed using qPCR with six serial 4-fold dilutions of pooled cDNA (1:5), followed by melt-curve analysis and determination of amplification efficiency from standard curves, in accordance with MIQE guidelines (Bustin *et al.*, 2009).

Several published primer sets did not meet performance criteria, due to either low amplification efficiency or evidence of genomic DNA amplification. In particular, the *VRN2* primer exhibited a small difference between primer melting and hairpin temperatures and amplified genomic DNA, while the *VRN3* primer also amplified genomic DNA and the ubiquitin primer showed low efficiency. To address these issues, alternative primer sets for *VRN2* and *VRN3* were designed using Primer3 (Untergasser *et al.*, 2012) to span exon–exon junctions, produce amplicons of 130–180 bp, and minimize primer dimer and hairpin formation. Additional published primers for *VRN2* (Dubcovsky *et al.*, 2006), *VRN3* (Yan *et al.*, 2006), and the housekeeping gene *Tef-1α* (Giménez *et al.*, 2011) were also evaluated, and the best-performing primer sets were selected. Details of all primers used in this study are provided in Supplementary Table S1.

### Verification of CAMP predictions

#### Model set-up and operation

The CAMP model was coded into a Python script that is available at https://github.com/HamishBrownPFR/CAMP/blob/master/CAMP.ipynb. A formal description of the code and parameterization scheme is given in the Supplementary Appendixes S1, S2. The FLN developmental phenotypes measured for each genotype (‘CAMP model general description’) were used to derive the *VRN* expression parameters needed for CAMP. Each of the treatments was simulated using CAMP with its corresponding daily temperature and Pp, so its predictions of *VRN* gene expression could be compared with those observed. The script running the CAMP code and producing the graphs displayed in this paper can be viewed at https://github.com/HamishBrownPFR/CAMP/blob/master/Tests/CAMPCETests.py.

#### Comparing CAMP predictions with observed *VRN* gene expression

Expression (qpcr*_v_*_,*i*_) of each gene (*v*) at each observation date (*i*) was normalised relative to EF1α (qpcr_ef,i_) and TA54227 (qpcr_ta,i_) expression for the same sample to provide relative levels of expression:


(5) $$\mathrm{qpc}r_{\mathrm{ref},i}=(\mathrm{qpc}r_{\mathrm{ef},i}+\mathrm{qpc}r_{\mathrm{ta},i})/2$$

(6) $$\mathrm{ObsRelEx}p_{v,i}=2^{-(\mathrm{qpc}r_{v,i}-\mathrm{qpc}r_{\mathrm{ref},i})}$$

To facilitate comparisons of the relative activity of individual genes (*v*), they were further normalized relative to maximum expression for *v* to give their relative activity (ObsRelAct*_v_*_,*i*_) on a scale from 0–1:


(7) $$\mathrm{ObsRelAc}t_{v,i}=\mathrm{ObsRelEx}p_{v,i}/\mathrm{ObsRelEx}p_{v,\max}$$

where ObsRelExp*_v_*_,*i*_ represents each observation (*i*) of ObsRelExp*_v_* and ObsRelExp*_v_*_,max_ is the maximum observed value of ObsRelExp*_v_*.

The Camp model predicts Vrn expression in relative amounts that are not directly comparable with ObsRelAct*_v_*. To facilitate comparison daily predictions of Vrn gene activity (camp*_v_*_,*i*_) were normalized relative to the maximum expression predicted across all treatments (camp*_v_*_,max_):


(8) $$\mathrm{PreRelAc}t_{v,i}\;=\;\mathrm{cam}p_{v,i}/\mathrm{cam}p_{v,\max}\times \mathrm{ObsRelAc}t_{v,\mathrm{cmax}}$$

Where ObsRelAct*_v_*_,cmax_ is the maximum observed relative activity for the genotype (c) under consideration. This multiplication is included to scale the predicted expression over the same range as the observations to further facilitate comparisons. Note: for *VRN1*, camp*_v_* is the sum of persistent cold Vrn1 and VrnB which are treated separately in the model (Supplementary Appendix S1) but assumed to represent the same *VRN* expression product measured in the plant.

These comparisons do not provide validation of the CAMP model as the data are not sufficiently independent from the model, but also that CAMP is not coded to predict absolute gene expression. Rather, it predicts relative accumulated effects of gene activity on developmental progress. While this is not directly comparable with observations of gene expression, comparison of modelled (Equation 8) and observed (Equation 7) time courses of relative activity are valid for assessing the mechanisms assumed in CAMP relative to temporal gene expression.

## Results

### Genotypic differences in developmental phenotype

**Fig. 4.**
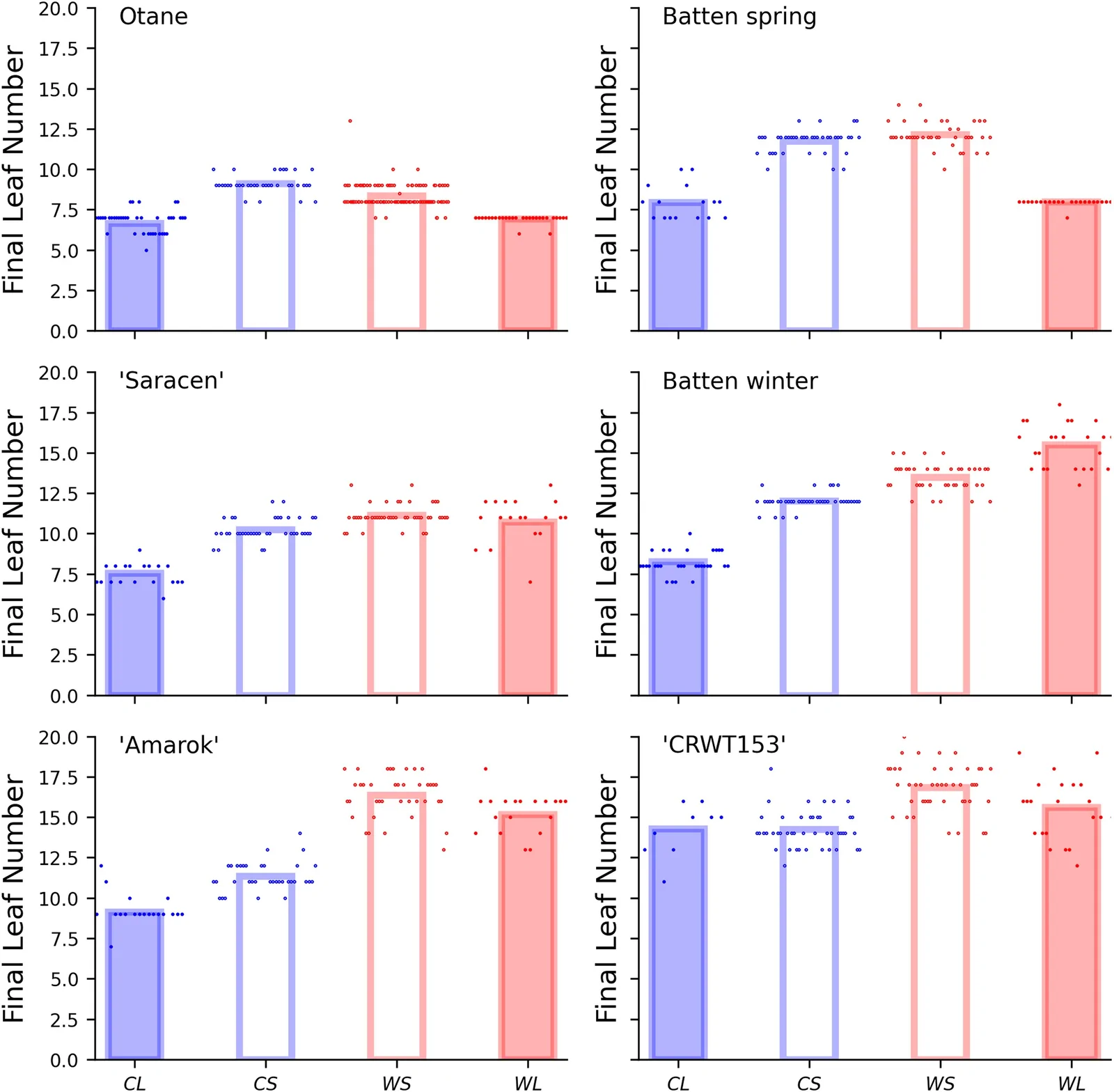
Final leaf number of different wheat genotypes grown in controlled environments under long (filled bars) or short (open bars) photoperiod, in combination with cold (blue) or warm (red) temperature treatment during the emerging phase. Treatments are denoted as CL (cold + long photoperiod), CS (cold + short photoperiod), WS (warm + short photoperiod), and WL (warm + long photoperiod). Points show individual plant observations and bars show treatment means.

The FLN values recorded under each environment treatment are shown in Fig. 4 and the developmental phenotypes derived for each genotype (using the scheme demonstrated in Fig. 2) are displayed in Table 2. There was a broad range in MinLN from 6.65 leaves for Otane up to 14.27 leaves for ‘CRWT153’. The PpLn ranged from no significant response in ‘CRWT153’ to a value of ∼3.8 leaves for the Batten pair. Of the winter types ‘Amarok’ had a significant CvLN of 5.02 but no significant PvLN. Alternatively, Batten winter had no significant CvLN but a PvLN of 5.75.

**Table 2.** Developmental parameters for wheat genotypes derived from FLN observations conducted in controlled environment conditions

	MinLN	PpLN	*P*	CvLN	*P*	PvLN	*P*
Otane	6.65	2.46	***	0	ns	0.29	ns
Batten spring	7.95	3.8	***	0.39	ns	−0.34	ns
‘Saracen’	7.55	2.71	***	0.87	ns	2.29	ns
Batten winter	8.28	3.75	***	1.47	ns	5.75	***
‘Amarok’	9.14	2.21	***	5.02	***	1.04	ns
‘CRWT153’	14.27	−0.03	ns	2.60	ns	−1.25	***

Probability (*P*) levels are from *t*-tests of final leaf number data between the two treatments used to calculate the sensitivities; α = 0.05 (***P<0.001; **P<0.01; ns, *P*>0.05). CvLN, cold vernalization sensitivity; MinLN, minimum final leaf number; PpLN, photoperiod sensitivity; PvLN, photoperiod vernalization sensitivity.

### The Haun stage timing of key phenological events

Fig. 3 shows the total organ number against HS for each treatment. The HS where observed organ number deviates from the basic vegetative rate represents VI^HS^ and the HS where observed organ number reaches a maximum represents TS^HS^. The positions of the observed and derived VI^HS^ and TS^HS^ correspond closely for all treatments providing verification for the scheme proposed to derive these values (see Supplementary Appendix S2).

**Fig. 5.**
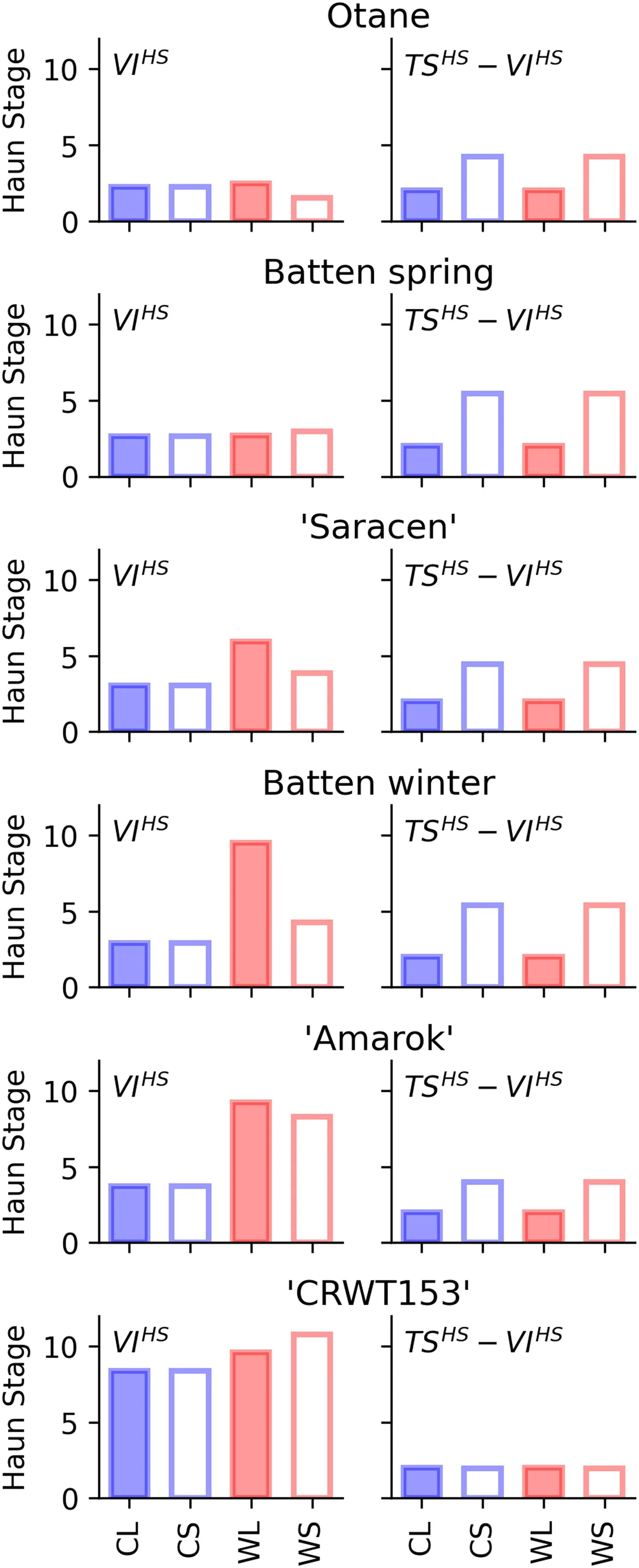
The Haun stage timing of vernalization induction (VI^HS^) and the duration of the early reproductive phase from VI^HS^ to the Haun stage timing of terminal spikelet (TS^HS^ - VI^HS^) of different wheat genotypes grow in controlled environment under long (solid fills) or short (hollow fills) photoperiod with (blue) and without (red) cold temperature treatment of 100 d at 1 °C prior to imposing photoperiod treatments. Treatments are denoted as CL (cold + long photoperiod), CS (cold + short photoperiod), WS (warm + short photoperiod), and WL (warm + long photoperiod).

From this analysis it is useful to compare the duration from VI^HS^ to TS^HS^ for different treatments (Fig. 5). For the cold treated plants, VI^HS^ = 2.0 for the two spring genotypes, 3.0 for ‘Saracen’ and Batten winter, 4.0 for ‘Amarok’, and 8.0 for ‘CRWT153’, and this was unaffected by photoperiod treatment. Under warm treatments the VI^HS^ of the spring genotypes was the same as under the cold treatments, confirming the assumption that spring cultivars express *VRN1* at high rates regardless of cold treatment. However, the VI^HS^ of the winter genotypes were delayed with warm treatments, and there was an interaction with photoperiod as to the extent of this (Fig. 5). Specifically, Batten winter showed the largest delay with VI^HS^ of 10 for WL treatment, but a much smaller delay with a VI^HS^ of 4.0 for the WS treatment, indicating shorter photoperiod causing less delay. ‘Amarok’ also showed a similar pattern, with substantial delay in VI^HS^ to 9.0 under WL and 8.0 under WS conditions. ‘CRWT153’ had a slight delay for the warm treatments, but with slightly more delay under long photoperiod than under shorter photoperiod, a pattern different from other genotypes. ‘Saracen’ also showed a delay in VI^HS^ to 6.0 under WL and 4.0 under the WS.

For the duration from VI^HS^ to TS^HS^, all the genotypes responded to photoperiod similarly, except ‘CRWT153’, which showed no photoperiod response (Fig. 5). Regardless of C or W treatments, the duration was reduced to minimum under L and maximized under S. Batten winter and spring had the same photoperiod response, consistent with their allelic variations (Table 1). Otane and ‘Amarok’ also had very similar response to photoperiod. These results verify the assumption that response to cold temperature ended at vernal induction.

### Observed gene expression over the development cycle of different genotypes in different environments

**Fig. 6.**
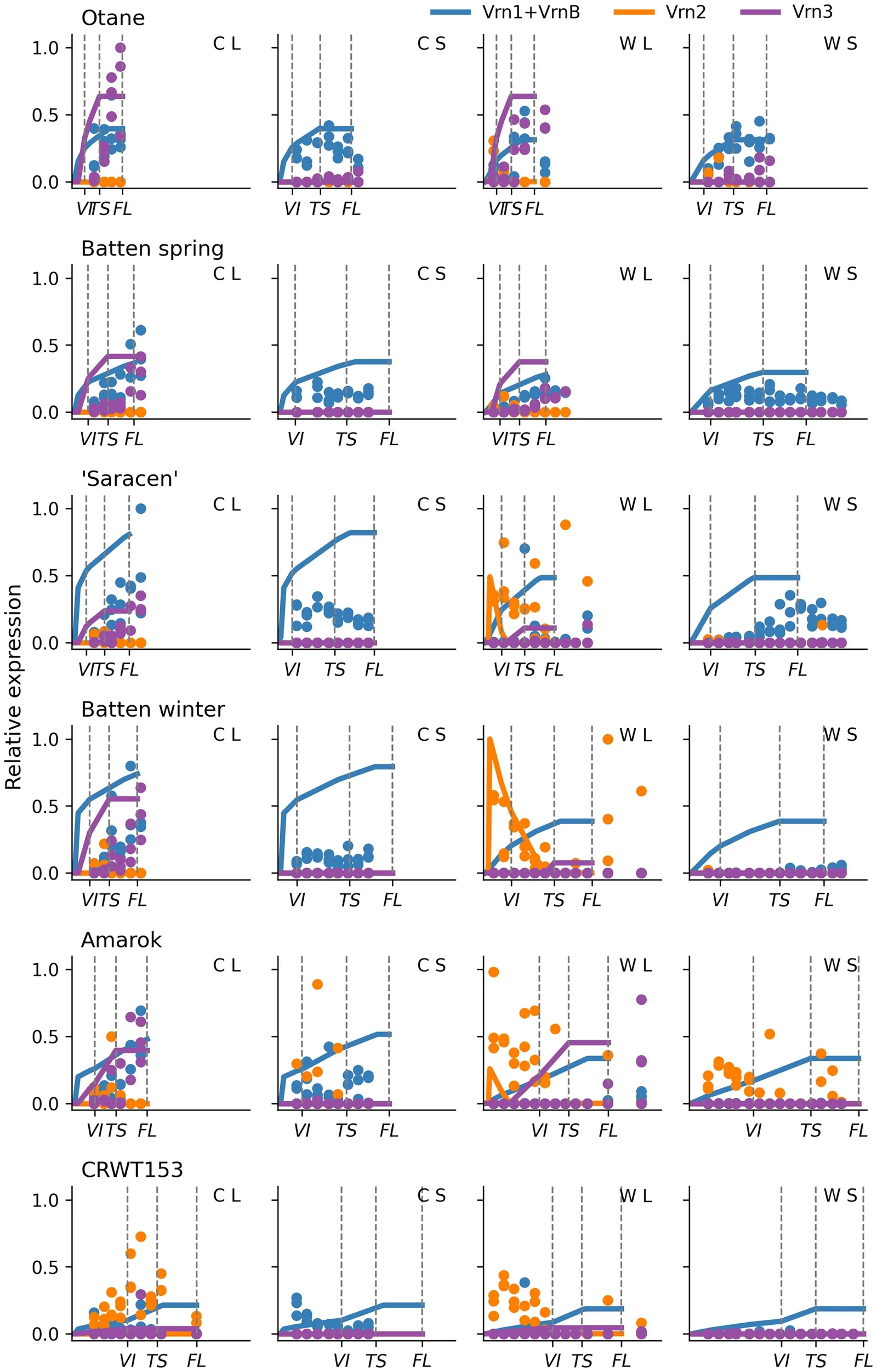
Relative expression of *VRN* genes in six wheat genotypes (arranged from lowest to highest minimum final leaf number, MinLN) grown under contrasting temperature and photoperiod conditions. Panels show time courses of relative gene expression for individual genotypes grown following cold (C) or warm (W) temperature treatment during the emerging phase, and subsequently under long (L; 16 h) or short (S; 8 h) photoperiod. Treatments are denoted as CL, CS, WL, and WS. The *x*-axis represents accumulated thermal time (base temperature = 0 °C), with vertical dashed lines indicating the timing of vernal induction (VI), terminal spikelet (TS), and flag leaf appearance (FL). The *y*-axis shows relative gene activity scaled between 0 and 1. Lines represent gene activity predicted by the CAMP model, and points represent observed foliar gene expression measured by RT-qPCR. Colours indicate genes: Vrn1+VrnB (blue), Vrn2 (orange), and Vrn3 (purple).

By first considering observed expression for the cold, long-day treatments (Fig. 6), the earliest genotypes (Otane, Batten spring, and ‘Saracen’) started with *VRN1* up-regulated in their leaves, which is soon followed by up-regulation of *VRN3* and further up-regulation of *VRN1*. Considering progressively later genotypes, the expression of *VRN2* extends for increasing periods before up-regulation of *VRN1*, down-regulation of *VRN2*, up-regulation of *VRN3*, and further promotion of *VRN1*. In the case of ‘CRWT153’, there is limited up-regulation of *VRN1* and *VRN3* and protracted up-regulation of *VRN2*.

Next consider the warm, long-day treatments (Fig. 6), where the spring genotypes (Otane and Batten spring) quickly up-regulate *VRN1*, down-regulate *VRN2*, up-regulate *VRN3*, and further up-regulate *VRN1* much the same as in the cold, long-day treatment. However, the winter genotypes show prolonged up-regulation of *VRN2* when no cold treatment is applied. The time of VI^HS^ corresponds to the down-regulation of *VRN2* but most of the winter genotypes do not display substantial up-regulation of *VRN1* or *VRN3*.

Next, consider the cold, short-day treatments (Fig. 6) where *VRN1* was up-regulated throughout development but remained at relatively static levels. Finally, consider the warm, short-day treatments (Fig. 6), where *VRN1* is up-regulated throughout for the spring genotypes, but only up-regulated after VI^HS^ for ‘Saracen’ or not up-regulated at all for the winter genotypes.

### Comparison of model predictions

Figure 6 also provides a comparison of the time course of gene activity predicted by CAMP relative to that observed for each of the six genotypes grown in each of the environmental treatments. The observed patterns of gene expression were in general agreement with what is conceptualized in the CAMP model where cold temperatures up-regulate Vrn1 in winter genotypes, but Vrn1 is up-regulated in spring genotypes regardless of temperature treatment; Vrn2 expression was limited to situations where Vrn1 expression was low under long photoperiods; Vrn3 expression was limited to situations where Vrn2 expression was low under long photoperiods. However, there are also situations where the observed and predicted Vrn expression differ. For example, after vernal initiation when Vrn2 is supressed, CAMP assumes that genotypes should display up-regulation of Vrn3 under long Pp, but this is not reflected in many of the observed patterns (Fig. 6).

The CAMP model tends to predict an up-regulation of relative VrnB+Vrn1 expression earlier than was observed for *VRN1* (Fig. 6). Under S conditions, it predicts a continued up-regulation of VrnB+Vrn1 where observed values of *VRN1* remained relatively static. For winter genotypes in W treatments, the model predicts an up-regulation of VrnB+Vrn1 where little *VRN1* was observed. A similar pattern was evident for *VRN3* where CAMP predicted its up-regulation earlier than observed and predicted substantial up-regulation for winter genotypes under W treatments when none was observed. By contrast, CAMP tended to predict Vrn2 down-regulation too early, and observations of *VRN2* were often made when the model predicted activity would be nil.

**Fig. 7.**
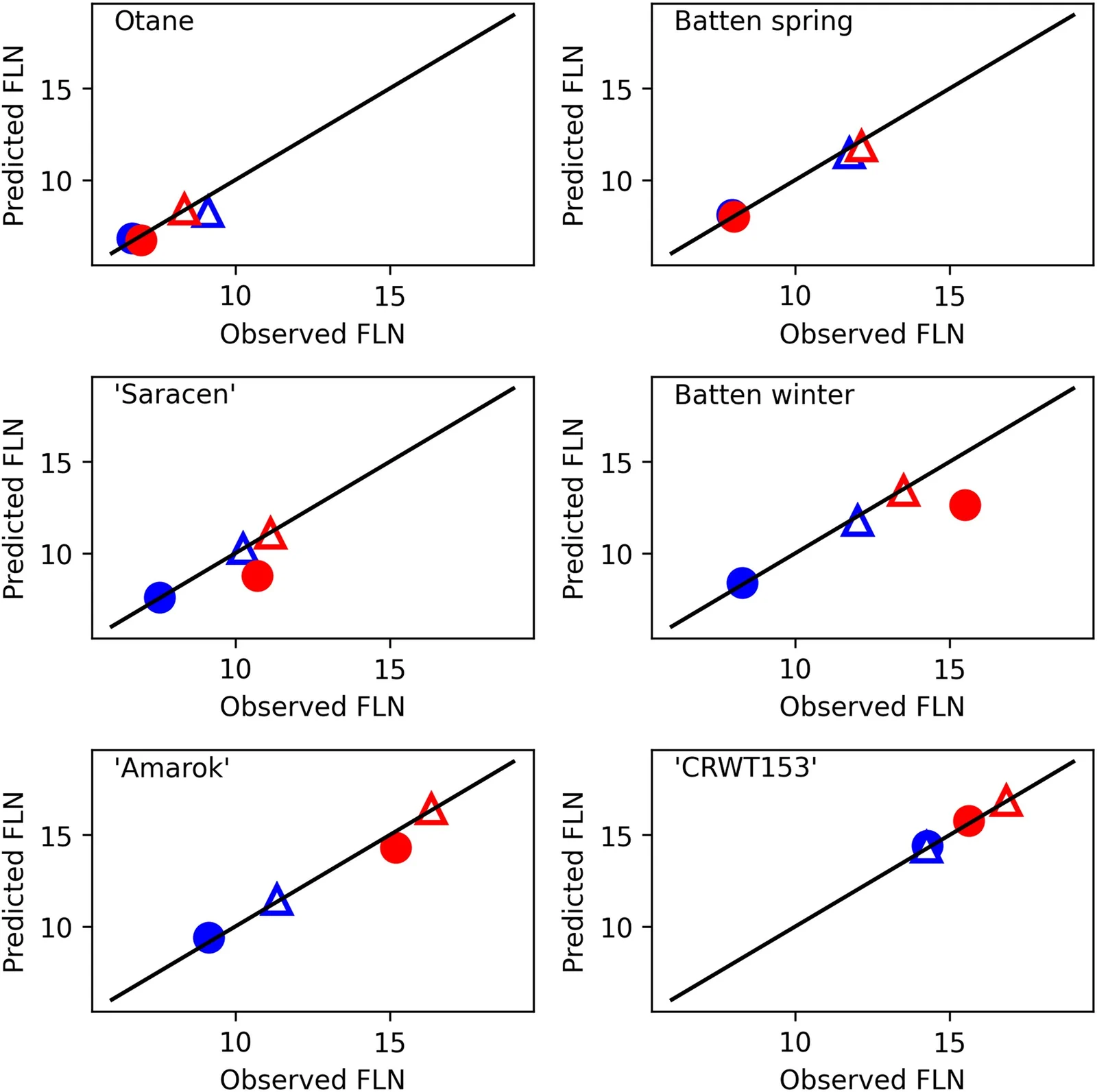
Comparison of observed and predicted final leaf number (FLN) for six diverse genotypes of wheat grown under 8 (open triangles) and 16 h pp (closed circles) with (blue) and without (red) cold temperature treatment during the emerging phase.

There was very good agreement between the FLN observed in each treatment and those predicted by the CAMP model (Fig. 7). This should not be considered a validation of the model as the observed data have been used in deriving and testing the model. However, it provides important verification that the phenotyping scheme, method for deriving CAMP Vrn expression rates, and the numeric implementation of the conceptual model can reproduce the broad range of FLN values created with a diverse set of genotypes and environmental combinations. In short, the phenotyping scheme and CAMP model provide a valid framework for quantifying genotypic differences and estimating how these will interact with environment to influence anthesis behaviour.

## Discussion

### Phenotyping method

The FLN-based phenotyping method proposed in this paper (in the section ‘Quantification of developmental phenotypes’) provides a useful approach for characterizing the developmental responses of wheat genotypes and providing data for determining parameters for the CAMP model for predictions of cereal phenology. It is expected that these phenotypes will be stable across different environments and so provide useful traits for genetic analysis of anthesis. The MinLN phenotype provides a quantitative representation of the genotypes earliness *per se*, which has a substantial range measured in the genotypes tested from 6.55–14.27 leaves. The CvLN provides a measure of the genotype sensitivity to cold temperatures during the vegetative phase. The PvLN and PpLN provide a measure of the photoperiod sensitivity of a genotype during the vegetative and early reproductive phases, respectively. Separating responses during the vegetative phase into cold and photoperiod responses and distinguishing photoperiod response between the vegetative and early reproductive phases is important. This is because photoperiod responses can work in opposite directions during these two phases and most other models are confounded because they fail to account for this. The phenotyping scheme proposed here demonstrates traits that related to gene expression controlling earliness *per se*, cold-temperature response (*VRN1*), short-day vernalization (*VRN2*) and photoperiod sensitivity (*PPD*).

Alleles of the major developmental genes have been determined for each of the genotypes used in this study (Table 1). However, there is substantially more variation in the phenotypes (Table 2) than can be related to these alleles alone. Other genes up- and downstream of the main control loop will also have an important role in the developmental phenotype. However, having a set of stable and unconfounded phenotypes provides a useful tool for genetic studies that aim to isolate and elucidate the role of different genes in wheat development.

### The link between *VRN* gene expression and developmental phenotype

The CAMP model was developed to provide a quantitative framework linking genotype to developmental phenotype for a given environment through the expression of key development genes. This model was used to explore the link between genotype (Table 1), observed developmental phenotypes (Fig. 4; Table 2), developmental physiology (Figs 3, 5) and foliar gene expression (Fig. 6). The FLN-based phenotypes (Table 2) are an easily observable outcome of the timing of VI^HS^ and TS^HS^, which are a less easily observable outcome of the expression of developmental genes. The gene expression depends on both the genetic makeup of a wheat plant and the temperature and photoperiod conditions it has grown through. The FLN-based phenotypes provide a relatively easy method of assessing the developmental phenotype, which is important for large scale genomic studies. The results presented have shown the relationship between FLN and the effects of cold and photoperiod on VI^HS^ and TS^HS^, assumed in the CAMP model, to be correct over a diverse range of genotypes (Fig. 3). As such, the remainder of the discussion is focused on the relationship between genotype, gene expression, and the timing of VI^HS^ and TS^HS^.

#### *VRN* gene expression and timing of vernal induction

The observed and predicted patterns of *VRN* gene expression under cold, long day conditions (Fig. 6) are consistent with the molecular models of foliar interaction of these genes (Chen and Dubcovsky, 2012). Early cold treatments had a range of effectiveness for the winter genotypes shown in both the extent of early *VRN2* and *VRN1* expression (Fig. 6) and the timing of VI^HS^(Fig. 5). The genetic control for this variation in response to cold temperature treatments is unknown, but it may relate to previously identified earliness *per se* loci (Sukumaran *et al.*, 2016).

The lack of foliar *VRN1* expression in the clear presence of *VRN2* down-regulation in the warm, long-day treatment is consistent with the suggestion of Dubcovsky *et al.* (2006) of the existence of an alternative *VRN2* repressor and with the results of Chen and Dubcovsky (2012) that foliar expression of *VRN1* is not essential for reproductive transition. The patterns of foliar *VRN* gene expression are consistent with the *VRN-A1* and *VRN-B1* alleles (Table 1). The lack of up-regulation of foliar *VRN1* in the winter genotypes suggests that the timing of VI^HS^ is controlled by apical expression of *VRN1* in combination with a *VRN3* protein signal from the leaf. Up-regulation of *VRN3* was not observed in the winter genotypes in this treatment, nor was it observed in many of the short-day treatments, although it is considered to be an essential element of the transition to reproductive development (Chen and Dubcovsky, 2012). Our results suggest that very low levels of foliar *VRN3* expression (below the detection range of real time qPCR) are required to signal floral transition in the apex.

Foliar *VRN1* was up-regulated from the first leaf throughout development for the cold, short-day treatment but remained at relatively static levels because *VRN3* was not up-regulated (Fig. 6). Comparing the cold, long-day treatment with the cold, short-day treatment, there is no difference in the time of VI^HS^ (Fig. 5) yet there are considerable differences in the patterns of foliar *VRN2* and *VRN3* expression (Fig. 6). This further supports the suggestion that the timing of VI^HS^ is controlled largely by *VRN1* expression in the apex and that foliar expression of *VRN* genes has little bearing, assuming a low expression of *VRN3* occurs.

In the warm, short-day treatments (Fig. 6), foliar *VRN1* is up-regulated throughout for the spring genotypes, but only up-regulated after VI^HS^ or not up-regulated at all for the winter genotypes. This further supports the notion that the timing of VI^HS^ is controlled largely by *VRN1* expression in the apex, and only a weak *VRN3* signal is required from the leaf. In the warm, long-day treatments, VI^HS^ occurs last, and genotypes where this is substantially later than under cold treatments (i.e. the winter genotypes) all showed prolonged foliar expression of *VRN2*. In all these cases, *VRN2* was eventually down-regulated, which allowed low levels of *VRN3* expression and the occurrence of VI^HS^.

Taken together, these results suggest that *VRN1* expression in the apex is mostly responsible for triggering VI^HS^, but foliar expression of *VRN2* will keep *VRN3* expression sufficiently low to prevent VI^HS^ until it is all down-regulated. When apical *VRN1* is up-regulated early, as in the cold treatments and the spring genotypes under warm treatments, the pattern of *VRN1* up-regulation will be passed to the leaves that develop from the apex, and this will hold *VRN2* expression low and allow VI^HS^ to occur. However, where *VRN1* is not up-regulated early, some mechanism other than *VRN1* repression of *VRN2* is required to unlock the *VRN3* signal from the leaves. Such mechanisms are likely to include photoperiod and circadian regulatory pathways acting upstream of the VRN genes. These have been demonstrated to influence flowering responses in cereals and related grasses, including *Brachypodium* (e.g. Woods *et al.*, 2019). Future improvements may be made to the CAMP model by explicitly modelling expression of *VRN* genes in both foliar and apical sites and further investigating the quantitative link between absolute levels of gene expression and changes in apical morphological behaviour. This would enable more direct comparison between observed and modelled outcomes and further improvement of our understanding of the link between genetics and phenological development.

#### *VRN* gene expression, developmental phenotypes and the timing of terminal spikelet

Once the crop reaches VI^HS^, the role of foliar gene expression becomes clearer, with a clear link between the expression of foliar *VRN3* and the duration from VI^HS^ to TS^HS^, which is directly related to PpLN phenotype. ‘CRWT153’ had zero PpLN (Table 2) and this was explained because it did not appear to up-regulate *VRN3* under either long or short photoperiod conditions (Fig. 6). The Batten genotypes had the greatest PpLN (Table 2; Fig. 4). This was because they did not express detectable levels of *VRN3* under short-day conditions and there was a large extension of the duration from the VI^HS^ to the TS^HS^ phase (Fig. 5). Otane and ‘Amarok’ expressed some *VRN3* under short-day conditions that would have caused some promotion of apical *VRN1* (Yan *et al.*, 2006), giving a smaller difference in FLN between long and short photoperiod treatments and a smaller PpLN (Table 2). The lower PpLN of Otane and ‘Amarok’ corresponded with allelic variation in *PPD-D1* (Table 1) but the genetic basis for the zero PpLN in ‘CRWT153’ is currently unknown.

The PvLN phenotype was relevant to the winter types (Batten winter, ‘Saracen’, and ‘Amarok’, Table 1) and its molecular cause can be clearly seen by comparing *VRN* gene expression under warm conditions (Fig. 6). Batten winter showed the largest PvLN (Fig. 4) corresponding to substantial foliar *VRN2* expression and no *VRN1* or *VRN3* expression under warm, long-day conditions. Under warm, short-day conditions, Batten winter did not express foliar *VRN2*, and VI^HS^ was substantially advanced (Fig. 5). By contrast, ‘Amarok’ had a small PvLN, expressed foliar *VRN2* under both L and S treatments, and showed a smaller reduction in VI^HS^. The difference in *VRN2* expression between Batten winter and ‘Amarok’ was probably caused by differences in the *PPD-D1* allele (Table 1) which sits upstream of *VRN2* expression (Mulki and von Korff, 2016). This shows another advantage of using the controlled environment phenotyping method described here, where the confounding effects of photoperiod and vernalization responses were clearly separated. The non-vernalized winter genotypes did not up-regulate *VRN1* under either short or long photoperiod after *VRN2* was down-regulated, which is consistent with the results of Dubcovsky *et al.* (2006).

The CvLN phenotype describes vernalization response to cold temperature, and ‘Amarok’ was the only genotype to show a significant response for this phenotype (Table 2) because of an increase in VI^HS^ going from cold short-day to warm short-day conditions (Fig. 5). ‘Amarok’ was also the only genotype to show substantial up-regulation of *VRN3* under short-day conditions (Fig. 6). Other genotypes that showed response to cold under long-day conditions (Batten winter and ‘CRWT153’) did not show response to cold (Fig. 5) or up-regulate *VRN3* under short-day conditions (Fig. 6). This shows a co-requirement for up-regulation of both *VRN1* and *VRN3* to achieve a vernalization response and identifies the photoperiod response of the *VRN3* gene as an important modulator of vernalization response.

### Concluding remarks

The data presented by Brooking and Jamieson (2002) and the analyses conducted by Brown *et al.* (2013) on the environmental responses of, and relationships between, FLN, TS^HS^, and VI^HS^ for the Batten near isogenic pair were replicated in the current work. The original CAMP conceptual model (Brown *et al.*, 2013) has been modified to better capture how expression of *VRN1*, *VRN2*, and *VRN3* influences development progress. The analysis presented here showed that the responses and relationships hold for a broad range of phenotypes demonstrating the versatility of the phenological framework in the modified CAMP model. Comparison of the relationships between environmental conditions, *VRN* gene expression, and the timing of key stages of apical development are consistent with those predicted by the new CAMP model. This supports its use to provide stable phenotypes for characterizing wheat genotypes. Specifically, exposure to cold temperature at early stages of development (before HS 2.0) up-regulated *VRN1* and accelerated the onset of VI^HS^ in winter wheat types. *VRN1* was up-regulated and VI^HS^ occurred early in spring wheat types regardless of the temperature conditions encountered. *VRN*2 and *VRN3* were only up-regulated under long photoperiod for most cultivars. The up-regulation of *VRN1* was accompanied by a down-regulation of *VRN2*. FLN-based phenotypes, assessed with a factorial of plus and minus early cold treatment and long and short photoperiod, provide unconfounded phenotypes that will be useful in large scale genomic studies. The CAMP model also provided a useful framework for reconciling the complex linkage between genotype, gene expression, apical physiology, and the resulting G×E patterns observed in FLN in wheat. The APSIM wheat model (Brown *et al.*, 2018) has been upgraded to include the CAMP phenology model, and a full description of this process is given in an accompanying paper (Wang *et al.*, 2026).

## Supplementary Material

erag278_Supplementary_Data

## Data Availability

All the data and scripts used to analyse data and produce graphs as well as CAMP model code are publicly available on the GitHub repository: https://github.com/HamishBrownPFR/CAMP/.

## Supplementary data

The following supplementary data are available at *JXB* online.

Table S1. PCR primers used in this study.

Appendix S1. Full description of the Cereal Anthesis Molecular Phenology (CAMP) model algorithms.

Appendix S2. Description of parameterization scheme for deriving genotype specific coefficients for CAMP.

Appendix S3. Observations of diurnal gene expression patterns.
